# The Utility of Preclinical Models in Understanding the Bone Health of Transgender Individuals Undergoing Gender-Affirming Hormone Therapy

**DOI:** 10.1007/s11914-023-00818-2

**Published:** 2023-09-14

**Authors:** Varun S. Venkatesh, Tian Nie, Jeffrey D. Zajac, Mathis Grossmann, Rachel A. Davey

**Affiliations:** 1 Department of Medicine, Austin Health, The University of Melbourne, Heidelberg, Victoria 3084 Australia; 2 Department of Endocrinology, Austin Health, Heidelberg, Victoria 3084 Australia

**Keywords:** Transgender, Gender-affirming hormone therapy (GAHT), Bone microstructure, Bone strength, Preclinical rodent models

## Abstract

**Purpose of Review:**

To summarise the evidence regarding the effects of gender-affirming hormone therapy (GAHT) on bone health in transgender people, to identify key knowledge gaps and how these gaps can be addressed using preclinical rodent models.

**Recent Findings:**

Sex hormones play a critical role in bone physiology, yet there is a paucity of research regarding the effects of GAHT on bone microstructure and fracture risk in transgender individuals. The controlled clinical studies required to yield fracture data are unethical to conduct making clinically translatable preclinical research of the utmost importance. Novel genetic and surgical preclinical models have yielded significant mechanistic insight into the roles of sex steroids on skeletal integrity.

**Summary:**

Preclinical models of GAHT have the potential inform clinical approaches to preserve skeletal integrity and prevent fractures in transgender people undergoing GAHT. This review highlights the key considerations required to ensure the information gained from preclinical models of GAHT are informative.

## Introduction

A transgender (or trans) person is someone who knows that their gender identity or gender expression is incongruent with their sex assigned at birth. This can lead to anxiety, depression and gender dysphoria. The term cisgender, or cis, refers to individuals for whom their gender identity matches their assigned gender at birth (e.g. cis female, cis male). Gender-affirming hormone therapy (GAHT) is a common treatment for gender dysphoria whereby patients receive sex steroids to align their physical features with their identified gender [1•]. Testosterone therapy is used to promote masculinisation in trans men (female-to-male), and estradiol therapy alone or in combination with anti-androgens is used to promote feminisation in trans women (male-to-female). In trans adolescents, many choose to undergo puberty suppression with gonadotrophin-releasing hormone agonists (GnRHa) to avoid the development of undesired pubertal characteristics of their natal sex prior to GAHT. GnRHa decreases circulating concentrations of sex steroids by downregulating GnRH receptors in the anterior pituitary thereby decreasing the secretion of luteinising (LH) and follicle-stimulating hormones (FSH). Pubertal suppression is commonly followed by GAHT to match the post-pubertal characteristics of their experienced gender. In adults, where puberty is already established, GAHT may be complemented by surgery to align physical characteristics with their gender identity. This review seeks to provide an overview of the current literature related to the effects of GAHT on bone health of trans people and the mechanistic action of sex steroid action in bone gained from preclinical models. Furthermore, we identify the current knowledge gaps within the field and suggest how preclinical models may be utilised to provide critical insight into possible changes that occur in bone structure and fracture risk following GAHT in trans people that cannot be gained by clinical studies.

## Sex Steroids and Bone Health 

Sex steroids are crucial for bone growth and peak bone mass accrual during puberty by regulating bone modelling. In adulthood, sex steroids are essential for the maintenance of bone by controlling bone remodelling. This is clearly illustrated by the low peak bone mass and bone loss that occurs in disorders of pubertal development or sex steroid deficiency in adulthood (e.g. post-menopausal women, hypogonadal men), respectively [2, 3]. Similarly, body composition is also regulated by sex steroids with men having proportionately more muscle mass whilst women have more fat mass [4, 5]; however, the actions of sex steroids on muscle and fat development are outside the scope of this review. GAHT is typically life-long, yet there is limited information regarding the long-term effects of GAHT in trans people on their skeletal integrity and fracture risk; this is an important knowledge gap which precludes evidence-based approaches to optimise bone health in this potentially vulnerable population.

### The Effect of GAHT on Bone in Trans Adolescents

Studies investigating the effect of puberty suppression and GAHT on bone in trans adolescents consistently show that blocking puberty with GnRHa significantly decreases areal bone mineral density (aBMD), volumetric BMD (vBMD) and modelling in trans girls and trans boys, preventing the attainment of peak bone mass compared to age-matched cis controls of the natal gender [6–8, 9••, 10–12]. Commencement of GAHT in trans girls and trans boys can partially reverse the bone loss associated with pubertal suppression but may not be of sufficient magnitude to restore BMD to levels of age-matched cisgender controls undergoing spontaneous puberty [12, 13], potentially placing these trans individuals at greater risk of fracture later in life. Crucially, the long-term impact of GnRHa/GAHT treatment in trans girls and trans boys on bone health and fracture risk is unknown and requires further investigation.

### The Effect of GAHT on Bone in Trans Adults

Despite the increasing number of recent studies investigating the impact of GAHT on bone in transitioning adults, confounding factors including deficiencies in study design (see the ‘Limitations of Clinical Studies Investigating Transgender Bone Health’ section) and conflicting results make accurate interpretation of the data difficult. Several reviews and meta-analyses discuss the clinical observations of GAHT on bone health in both trans women and trans men in depth [14–17, 18•] of which we provide an appraisal of the key findings below.

### The Effect of GAHT on Bone Health in Adult Trans Men

Evidence from meta-analyses of both cross-sectional and longitudinal studies indicates that in trans men GAHT has a neutral or slightly net positive effect on bone health compared to cis females measured by aBMD at the spine, total hip and femoral neck [15, 17, 18•]. Increases in total vBMD at the radius and tibia, cortical and trabecular thickness following a median of 3.5 years of GAHT measured by pQCT, HR-pQCT [19•] and histomorphometry [20] as well as increases in trabecular vBMD and cortical bone size but lower cortical vBMD following 10 years of GAHT in trans men compared to cis women have been reported [21]. Collectively, these data suggest that bone microarchitecture is not compromised by GAHT in trans men.

### The Effect of GAHT on Bone Health in Adult Trans Women

Data from systematic reviews and meta-analyses indicate that reports on the effects of GAHT on bone health of trans women are inconsistent [14, 15, 17]. Several cross-sectional studies show that GAHT negatively impacts bone health characterised by lower aBMD and vBMD and deteriorated bone microstructure compared to cis men [19•, 22, 23]. Conversely, there are reports from both systematic [15] and longitudinal [24–28] studies of higher aBMD in trans women undergoing GAHT. A more recent longitudinal study by Weipjes et al. report no changes in lumbar spine BMD in trans women after 10 years of GAHT [29]. These reports should be interpreted with careful consideration as aBMD is a 2D measurement and is calculated as bone mineral content (BMC) divided by bone area so that aBMD is inversely related to bone size. Measures of vBMD measured using high-resolution peripheral quantitative computed tomography (HR-pQCT) however provide 3-dimensional images that attempt to account for bone size differences and bone geometry. Recently, a cross-sectional study of trans women on GAHT for a median of 3.2 years reported lower total vBMD, cortical vBMD, thickness and increased porosity measured by HR-pQCT at the distal radius and distal tibia compared to cis male controls [19•]. In addition, trabecular bone volume/tissue volume (BV/TV) was lower in trans women accompanied by lower trabecular number (TbN) and trabecular thickness (TbTh). Further well-controlled longitudinal studies assessing bone microarchitecture using high-resolution imaging modalities are necessary to resolve the conflicting data regarding the effects of GAHT on bone health in trans women. It is also important to note that the majority of studies investigating the effects of GAHT on BMD in trans women discussed above made comparisons with a single control group of individuals with the same sex assigned at birth, i.e. cis males. However, given the well-documented sex differences in the skeleton between men and women, these data would be more informative if comparisons were also made to a control group of the desired sex, i.e. cis females.

### The Effect of GAHT on Fracture Risk in Trans Women and Trans Men

Currently, there are only two retrospective cohort studies determining fracture risk following GAHT in trans adults. Wiepes et al. showed that in 934 trans women aged ≥ 50 years on GAHT for a median of 19 years, fracture risk was higher (odds ratio = 1.90, 95% CI 1.32–2.74) compared to age-matched healthy cis men, with no apparent increase in fracture risk in trans men on GAHT for a median of 9 years compared to cis male controls [23]. The second study focused on 57 trans women who underwent gender-confirming surgery ≥ 2 years prior to study onset and were on GAHT for a median of 5 years. Fracture risk was assessed by DeFRA fracture risk algorithm (adapted from FRAX score) [30]. Low bone mass compared to cis males was noted in 40% of the participants, with 12% of trans women having an intermediate fracture risk and one with a high fracture risk. Of note, algorithms such as FRAX only allow sex, not gender as a variable with the data used to stratify patient data being derived from cisgender cohorts. As such these algorithms may not be able to accurately predict fracture risk in the transgender population and warrants further investigation.

## Limitations of Clinical Studies Investigating Transgender Bone Health

There are several limitations inherent in these studies of the effects of GAHT on bone health in trans men and trans women that are important to note. These range from observational design, small group sizes, ad hoc sample collection, lack of appropriate control groups and different treatment regimens. As GAHT is commonly individualised for each patient, there are often differences in the route of administration (i.e. transdermal, intramuscular, oral) as well as possible variations in circulating sex steroid concentrations due to patients electing to increase or decrease their prescribed dose of GAHT [31]. In trans adolescents, the data is further complicated by differences between patients in the duration of GnRHa treatment to suppress puberty prior to commencing GAHT to affirm to their desired gender. The appropriate control groups for studies in transgender people should be carefully considered to ensure accurate interpretation of the data. As raised in ‘The Effect of GAHT on Bone Health in Adult Trans Women’ section above, whilst most studies compare parameters between trans individuals and cis controls of their assigned sex at birth, we would argue that comparisons between trans individuals and cis controls of their desired gender would provide significant insight into the effects of GAHT.

A major confounding factor in studies investigating the effects of GnRHa/GAHT in trans girls and GAHT in trans women are reports from cross-sectional studies showing that trans girls and trans women have a lower BMD compared to age-matched cis males prior to the commencement of GAHT [10, 13, 32, 33] so that differences in BMD cannot be ascribed to the effects of GAHT with certainty. The reason for this is unclear but may be attributed to differences in lifestyle and exercise intensity between trans girls and boys [13] whilst trans women have been reported to have a lower muscle mass, body weight and serum vitamin D concentrations compared to cis men [28], possibly because of their lower participation in physical activity from a young age compared to cis male controls [34]. Differences in physical activity between trans individuals and cis controls are also likely to occur following GAHT as sex steroids have been shown to regulate voluntary activity in male and female mice [35–37], making accurate interpretation of the direct effects of GAHT on BMD bone challenging.

Since withholding GAHT is unethical, it is unlikely that randomised placebo-controlled clinical long-term trials investigating fracture risk in trans people will be conducted. Furthermore, measurements of bone microstructure and bone cell metabolism in trans people require an invasive bone biopsy and crucially, direct measurements of bone strength to precisely determine fracture risk are not possible in humans. As such, clinically relevant preclinical research in this area is of the utmost importance.

## The Use of Preclinical Rodent Models for Studying the Effects of GAHT on Bone

### Advantages

Rats and mice are effective models for investigating the effects of GAHT on bone cell activity, integrity and strength as many aspects of the regulation of bone are shared between mice and humans. The use of mice circumvents the need to collect invasive bone biopsies required for the high-resolution assessment of bone microstructure and bone cell activity, but most importantly, direct measures of elasticity and breaking strength as an indication of fracture risk following GAHT can be performed in mice, which are not possible in humans. In addition, the considerably faster remodelling rate of trabecular bone in mice (~ 2 weeks compared to 6–9 months in humans) enables increased research productivity [38]. Finally, preclinical mouse models lack cofounders inherent in clinical trials (i.e. socioeconomic factors, lower BMD of trans girls/women compared to cis boys/men prior to GAHT).

### Factors to Consider When Making Inferences from Preclinical Rodent Models to Transgender Bone Health

Clinically relevant findings related to effects of GAHT on bone health can be obtained from rodent data if the differences are fully appreciated [38]. A key difference between the skeletons of humans and rodent is their posture with the quadrupedal nature of rodents leading to differences in weight distribution and mechanical loading compared to bipedal humans. Additionally, longitudinal bone growth ceases following puberty in humans due to closure of the epiphyseal growth plate, whilst, in mice, the epiphyseal plate remains open and they continue to grow at a slow rate post-puberty [38, 39]. There is also a lack of evidence for osteonal cortical bone remodelling in mice and rats due to the absence of Haversian canals [38, 40, 41]. There is however the presence of blood vessels and intracortical pores in rodent bones, indicative of a more primitive form of Haversian remodelling [6, 38, 42] and more recently, the age-related increase in cortical porosity that occurs in mice has been attributed to de novo intracortical remodelling by osteon-like structures [43].

Of relevance to studying the effects of GAHT in rodents is the fact that they lack post-natal endogenous sex hormone-binding globulin (SHBG), which regulates circulating sex steroid concentrations as well as modulating the bioavailability of sex hormones by preventing their diffusion into target tissues [44]. As such, the absence of SHBG in rodents leads to large variations in their concentrations of circulating sex steroids thus making their accurate measurement challenging. The use of LC–MS/MS for determining circulating sex steroid concentrations is likely to assist in decreasing this variability as it is considered the ‘gold standard’ for such measurements due to its higher sensitivity and reproducibility than other methods such as ELISA and radioimmunoassays [45]. In addition, the selection of the strain and age of the rodent model should be carefully considered. BMD and the magnitude of bone loss due to oestrogen deficiency in ovariectomised rats and mice models differ between different strains [46–48]. It is also important to select an age of the rat or mouse model that accurately reflects the bone age in humans, i.e. pre-pubertal, before the increase in bone mass accrual associated with puberty, versus adulthood, when peak bone mass has been achieved. It is not possible to ascribe an optimal age to represent early puberty and adulthood that is appropriate for all studies involving mice or rats as the age of puberty onset and skeletal maturation in rodents is both sex and strain dependent [38, 49–52]. For example, however, in one of the most commonly used mouse strains for bone research, C57BL/6, the appropriate age to represent early puberty in females as measured by vaginal opening is approximately 28 days, whilst in males, it is 30 days as determined by balano preputial separation [51]. For adulthood, the recommended age is 16 weeks of age in both males and females when peak total body BMD has been reached [39]. It is also worth considering that since peak bone mass in humans is typically achieved in the early 20 s for females and late 20 s for males [53], the inclusion of a younger adult group of mice to model trans people who commence GAHT whilst ostensibly ‘adults’ without having reached peak bone mass would also yield valuable information (e.g. 12 weeks of age for C57Bl/6 mice [54]). By considering the morphological and developmental differences from humans whilst leveraging their physiological advantages, data generated from preclinical models allows for significant predictions related to transgender bone health that would otherwise not be possible.

## The Actions of Sex Steroids on Bone — What We Have Learned from Preclinical Rodent Models

Significant insight into the action of sex steroids in bone has been gained from castration, pharmacological and genetically modified rodent models which have been reviewed extensively [6, 55–57]. The data from these studies also offer important information pertinent to the effects of GAHT on bone health in transgender individuals. Here we summarise the data arising from these rodent models.

### Surgical Castration Models

Gonadectomy suppresses circulating sex steroid concentrations through the removal of the ovaries or testes and is a well-established model used to study the effect of sex steroids on bone health [58, 59]. As rodents do not synthesise sex steroids in their adrenal glands [60], the primary sex steroid source is from the gonads. Removal of sex steroids via gonadectomy in both males and females results in increased bone remodelling, leading to cortical and trabecular bone loss [47, 61, 62] and decreased bone strength [63–65].

### Treatment with Gonadotropin-Releasing Hormone Agonists (GnRHa)

GnRHa have been used in preclinical models to elucidate the effects of blocking sex steroid production on bone growth and accrual during puberty. Pubertal male mice treated with high doses of GnRHa to induce complete pubertal suppression exhibit a marked impairment in femoral cortical and trabecular bone acquisition analogous to that reported in gonadectomised male mice [66••]. Co-treatment with estradiol and GnRHa in pubertal female Sprague–Dawley rats following 8 weeks of GnRHa therapy alone was able to partially prevent the decrease in BMD of the femur and trabecular bone loss in the lumbar vertebrae [67]. Together, these data support the essential role of the sex steroids for optimal bone mineral accrual during puberty in both males and females and are consistent with the deleterious effects of GnRHa on the attainment of peak bone mass in adolescent trans males and trans females [66••, 67–69]. Other pharmacological agents such as androgen and selective oestrogen receptor modulators [70, 71] or aromatase inhibitors [72] are useful for identifying the roles of sex steroids in bone, however, lack the specificity required to elucidate effects on discrete tissues or cell types which is better offered by genetically modified mouse models.

## Genetically Modified Mouse Models to Investigate Sex Steroid Action in Bone

Numerous genetically modified mouse models have been generated to investigate the actions of sex steroids in regulating bone via their respective receptors within bone as well as other target tissues such as neurons [35, 73] and muscle [74]. Given the extensive data these models have yielded, we focus our discussion below on data obtained from global- as well as bone cell-specific ER and AR deletion mouse models.

### Genetically Modified Mouse Models to Investigate Estradiol Action via the Oestrogen Receptor (ER) in Bone

#### Global ER Knockout Mouse Models

A wealth of knowledge regarding the physiological actions of estradiol via the two DNA-binding-dependent receptors, ERα and ERβ, in bone of both males and females has been generated using single or double ER global knockout (KO) mouse models (reviewed by [57, 75]). Much of the data arising from the ERKO models are conflicting and challenging to interpret due to several confounding factors that are often inherent to genetically modified mouse models. For example, deletion of ERα in both male and female mice disrupts negative feedback to the hypothalamic-pituitary–gonadal axis (HPG) resulting in increased circulating concentrations of sex steroids which can act in bone via either the AR or ERβ [76]. Gonadectomy and treatment of ERα mice with ideally physiological replacement doses of estradiol are therefore required to accurately identify the specific effects of ERα deletion on bone [77]. The gene targeting approach also has the potential to influence the resulting phenotype. For instance, the ERβKO mouse line characterised by Sims et al. was null for ERβ with complete absence of the ERβ protein whilst other mouse lines reviewed by Khalid and Krum [78] have an ‘in frame deletion’ of the DNA-binding domain resulting in a mutant protein that may be capable of activating the non-DNA-binding-dependent pathways of ERβ action [79].

Despite these differences, taken together, the data generated from Global ERKO mouse models indicates that the regulation of bone by estradiol in males and females is predominantly mediated via the ERα, whilst in female bone, ERβ can partially compensate for ERα [77, 80]. ERα and ERβ also play crucial but opposing roles in the adaptation response of bone to loading with ERαKO mice having a lower and ERβKO having a higher response to mechanical loading respectively [81]. Signalling via the membrane bound ‘G protein-coupled oestrogen receptor’ (GPER-1) has been investigated more recently and may modulate bone growth through signalling in growth plate chondrocytes, though this effect was not seen using GPER-1 agonists [82, 83].

#### Bone Cell-Specific Deletion of the ERs

Further insight into estradiol action in bone has been achieved using the Cre/*lox*P system to target the deletion of ERα and ERβ to specific bone cell types to overcome the confounding effects of systemic changes in sex steroids observed in Global ERKO models (reviewed by [75, 78]). In female mice, deletion of ERα in osteoclasts decreases trabecular bone mass due to increased bone remodelling, but not cortical bone mass [84, 85] whilst there is no effect in males [85].

The action of estradiol via the ERα in osteoblast-lineage cells is dependent on sex and the stage of osteoblast differentiation. In osteoblast progenitors expressing paired-related homeobox protein 1 (*Prx-1*) and Osterix 1 (*Osx-1*), deletion of the ERα decreased femoral BMD in adult females due to a decrease in cortical thickness whilst trabecular BV/TV was unaffected [68]. In contrast, deletion of ERα in mature osteoblasts expressing type 1α1 collagen in females had no effect on trabecular or cortical bone mass [68]. It is therefore somewhat surprising that deletion of the ERα in osteoblasts at the later mineralisation stage of differentiation expressing osteocalcin (OCN) in female mice results in reductions in both femoral and vertebral cortical and trabecular BV/TV evident from 3.5 months of age, whilst reductions in trabecular BV/TV of male OCN-ERαKO mice were not observed until adulthood at 6 months of age [86]. In terminally differentiated osteocytes, expressing dentin matrix acidic phosphoprotein 1 (DMP-1), Windahl et al. showed that deletion of ERα decreased trabecular BV/TV exclusively in male mice [87] whilst in contrast, Kondoh et al. showed that osteocytic deletion of ERα led to trabecular osteopenia in female but not male mice [88]. To avoid the confounding effects of disrupting ERα signalling during development on the skeleton, Doolittle et al. inactivated ERα in osteocytes in adult mice using a tamoxifen-inducible ERαKO model [89] and showed a reduction in trabecular BV/TV in females due to reduced bone formation as well as increased periosteal and endocortical diameters, whilst males were unaffected. It is worth noting however that caution may be required when interpreting data from tamoxifen-inducible mouse models as there are conflicting reports of tamoxifen dosing regimens having documented effects on the skeleton [90, 91•] whilst others report minimal effects on bone [92].

Unlike the ERα, the action of ERβ in specific bone cells has not been extensively studied. In contrast to deletion of ERα in early osteoblast progenitors in females which only effects cortical bone [78], the deletion of ERβ in osteoblast progenitors expressing *Prx1* results in increased trabecular BV/TV although cortical bone is unaffected [93]. The effects of ERβ in osteocytes are complex being age, sex and site specific. Deletion of ERβ in mineralising osteocytes expressing OCN increased trabecular BV/TV in the vertebra of young male mice, whilst it was increased in adult males and unaffected in females. Similarly, osteocyte ERβ deletion reduced the cortical osteogenic response to mechanical loading in the proximal tibiae in young and old males, whilst the osteogenic response was increased in young but not adult females [94].

Collectively, the data from these bone cell specific ERα- and Erβ-KO mouse models suggest that the actions of estradiol in female bone is mediated via both osteoclast and osteoblast lineage cells with the protective effects of ERα on cortical bone to inhibit periosteal bone formation and endocortical bone resorption being mediated via osteoblasts and osteocytes, whilst in trabecular bone its via osteoclasts. The roles of ERα in males and ERβ in both males and females in specific cell types within bone are more complex and require further study.

### Genetically Modified Mouse Models to Investigate Androgen Action via the Androgen Receptor (AR) in Bone

#### Global ARKO Mouse Models

ARKO mouse models have been instrumental for distinguishing the actions of androgens mediated directly via the AR from those that arise from its aromatisation of testosterone to estradiol and action via the ERs. Several Global-ARKO mice have been generated in which the AR gene is deleted pre-natally. Despite differences in the region of the AR gene deleted and the genetic background of these Global-ARKOs, it is clear from their phenotype that the AR is essential for bone growth and accrual in male mice with male Global-ARKOs having smaller bones of reduced periosteal circumference, cortical thickness and trabecular BV/TV as a result of increased remodelling [95–98]. The effect of global ARKO deletion in females is less marked with small decreases in periosteal and medullary circumference whilst trabecular BV/TV is unaffected [96]. AR deletion does not impact longitudinal bone growth in either male or female Global-ARKOs, which supports the notion that longitudinal bone growth is predominantly regulated by ERα in both sexes [6, 99]. Tamoxifen-inducible global deletion of the AR in male mice either pre-pubertally at 4 weeks of age or post-pubertally at 10 weeks of age results in decreased total body BMD, cortical thickness and trabecular BV/TV suggesting that the AR is essential for bone growth and accrual during puberty and for cortical and trabecular bone maintenance in male adults [99].

#### Bone Cell-Specific Deletion and Over-expression Mouse Models of the AR

Significant insights into the mechanisms of AR action in male bones have been gained using several different genetically modified mouse models where, in osteoblasts at defined stages of their development, the AR gene has been deleted [95–98, 100, 101], over-expressed [102, 103] or replaced in Global-ARKOs [104]. Collectively, this large body of work shows that AR action in osteoblasts in male mice is dependent on their stage of maturation. In proliferating osteoblasts, the AR stimulates periosteal apposition of cortical bone and accrual of trabecular bone during growth [103], independent of androgen action in other tissues [104]. Post-puberty, the AR predominantly acts via the AR in mature and mineralising osteoblasts to maintain trabecular bone [100–102] by inhibiting bone resorption. This is mediated, at least in part, by decreasing the ratio of receptor activator of nuclear factor kappa-Β ligand (RANKL) to osteoprotegerin (OPG) expression by osteoblasts [104], whilst deletion of the AR specifically in osteoclasts has no effect [105]. AR action in mineralising osteoblasts and osteocytes in males is also important for the coordination of bone matrix synthesis with its subsequent mineralisation [100] and to protect against age-related loss of trabecular integrity [106]. In contrast to males, the effects of AR action in osteoblasts in females are modest [102, 103]. Collectively, these data indicate an essential role for the AR for pubertal bone mass accrual and for maintaining bone health post-puberty in males whilst its role in regulating bone in females is relatively minimal.

### Genetically Modified Mouse Models to Investigate Aromatase Action in Bone

Aromatase (Ar) KO mice are advantageous in understanding the contribution of aromatase mediated conversion of testosterone to estradiol in bone physiology. Similar to humans with life-long aromatase deficiency who exhibit osteopenia or osteoporosis [107], both male and female ArKO mice have decreased trabecular BV/TV and a sexually dimorphic pattern of high remodelling in females and low remodelling in males compared to sex-matched controls [108]. Treatment of young ArKO male and female mice with estradiol at 9 weeks of age has been shown to completely restore femoral BMD and trabecular BV/TV to control levels [109]. These data suggest an important role for oestrogen in the attainment of peak bone mass in female mice; however, further research is required to fully elucidate the importance of aromastase activity in preserving skeletal integrity.

## Circulating Concentrations of Sex Steroids Versus Their Local Concentrations Within Bone 

**Fig. 1:**
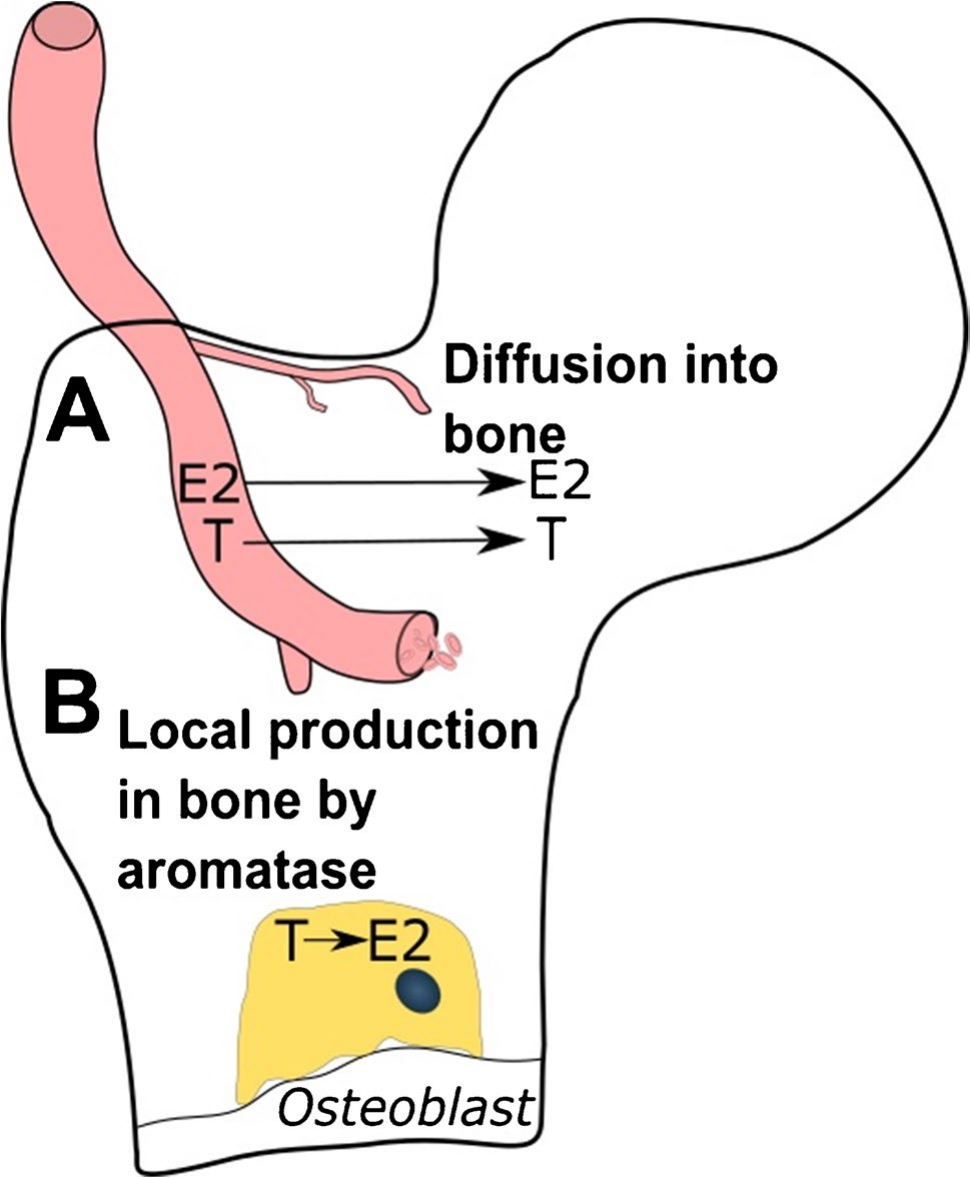
The concentration of sex steroids within bone is determined by (**A**) diffusion from the circulation into bone and by (**B**) the local synthesis of estradiol from testosterone by aromatase expressed by osteoblasts within bone

A major aspect of understanding the effects of GAHT on bone and fracture risk is to answer the long-standing question: ‘what is the contribution of the diffusion of sex steroids from serum into bone (Fig. 1A) versus the local synthesis of estradiol from testosterone by aromatase expressed by osteoblasts within bone (Fig. 1B) in preserving skeletal integrity?’ The answer to this question is clinically relevant since GAHT may have differential effects on circulating versus local sex steroid concentrations within bone. For example, whilst trans women on GAHT achieve physiological concentrations of serum estradiol, the negative feedback of estradiol on the HPG axis to reduce serum testosterone may result in insufficient substrate for the local synthesis of estradiol by aromatase within bone. Reductions in the concentrations of estradiol within bone would lead to increased bone remodelling and subsequent bone loss.

The importance of local aromatisation of testosterone to estradiol within bone to maintain its integrity is particularly evident in males. In men and mice, estradiol is thought to be the dominant sex steroid regulating bone resorption, with both testosterone and estradiol being important for bone formation [77, 110–112]. Interestingly, however, at least in male mice, the circulating concentrations of estradiol are 100–1000-fold lower than the concentrations required to activate the ER [113••] suggesting that the local concentrations of estradiol within bone must be substantially higher than the circulating concentrations in order to maintain skeletal integrity. Supportive evidence for a possible role of local aromatisation of testosterone within bone is provided by a prospective case study of 23 trans men naïve to GAHT and ovariectomy, who prior to treatment had estradiol concentrations comparative to the control group of cis women (*n* = 23). After 1 year of GAHT, testosterone concentrations increased by 15-fold (baseline mean ± SD 27 ± 12 ng/dl to 629 ± 225 ng/dl) and estradiol concentrations decreased 1.7-fold (baseline median (1st–3rd quartile) 54 (24–110) pg/mL to 31 (23–36) pg/mL). Despite the reduction in circulating estradiol concentrations, hip aBMD and trabecular vBMD increased compared to cis women [114]. These data suggest that the increase in circulating testosterone was able to compensate for the decrease in serum estradiol concentrations via activation of the AR within bone. Alternatively, the increase in circulating testosterone may have resulted in an increase in the local concentrations of testosterone within bone via diffusion which was subsequently aromatised to estradiol. Since both pathways are present, it is difficult to ascertain the contribution of each of these pathways in increasing BMD in these trans men. Evidence to support the local production of estradiol within bone by aromatisation is provided by reports of higher concentrations of estradiol within bone than in the circulation of orchidectomised, testosterone-treated male mice [44]. In addition, BMD is markedly increased in male mice that over-express aromatase in osteoblasts (Obl-Artg) compared to controls whilst no effect was observed in female Obl-Artgs [115]. The lack of effect in female Obl-Artgs was attributed to their low levels of circulating testosterone (the necessary substrate for aromatase to produce estradiol), with a subsequent increase in BMD observed following exogenous testosterone in these mice confirming this notion [115]. Taken together, it is therefore conceivable that following GAHT in trans males and trans females, the major determinant of skeletal integrity is likely to be estradiol produced locally within bone by aromatisation of testosterone, rather than by diffusion from the circulation into bone.

If this hypothesis is correct, we would predict that in trans men, the increase in circulating testosterone concentrations following GAHT would increase the concentrations of testosterone within bone which would be aromatised to estradiol. The resulting increase in estradiol concentration within bone would decrease remodelling thereby preserving bone volume, microstructure and strength compared to cis females which is consistent with reports or a neutral or slightly positive effect of GAHT on the bone health in trans men (‘The Effect of GAHT on Bone Health in Adult Trans Men’ section). In contrast, in trans women, we predict that estradiol treatment would decrease circulating testosterone concentrations due to negative feedback on the HPG axis, leading to a decrease in testosterone concentrations within bone, decreased aromatisation and estradiol concentrations resulting in increased remodelling, decreased bone volume and reduced strength compared with cis males. If this is the case, then it follows that GAHT-treated trans women, despite achieving circulating estradiol concentrations in the cis female range, will have insufficient estradiol concentrations within bone to maintain their health and prevent fracture, a risk not recognised by monitoring their circulating sex steroid concentrations, the current approach used to tailor GAHT. It is not possible to test this hypothesis in the absence of a bone biopsy pre- and post-GAHT in trans men, trans women and cis controls; however, preclinical mouse models are advantageous for answering these questions.

## Preclinical Mouse Models of Transgender Health

Whilst there is a growing body of literature utilising animal (predominantly rodent) models to study the effects of GAHT on the reproductive, cardiovascular and neurological systems, only two studies have focussed on bone to date (‘The Effects of GAHT on Bone in Preclinical Mouse Models of Female-to-Male Transition’ section). Although these studies have some limitations including a short duration of GAHT, the various methods used to measure circulating sex steroid concentrations (i.e. ELISA, immunoassay, LCMS/MS) with varying degrees of specify and sensitivity, various routes of administration (e.g. oral vs transdermal) and hormone formulation (e.g. beta-estradiol vs estradiol valerate or cypionate), important findings related to the effects of GAHT in these preclinical mouse models have been made which are summarised in Table 1. A possible candidate animal model not yet utilised for transgender research which may provide valuable information is the ovine model which unlike rodents, has the additional advantage of expressing endogenous sex hormone-binding globulin [116] and their utility for osteoporosis research in orthopaedics is clearly established [117]. In this section, we will critically review the findings from the two preclinical studies in bone by Goetz et al. [118] and Dubios et al. [119••] as well as identify the gaps in our current knowledge and the critical research questions that need to be addressed to better understand the effects of GAHT on bone health in trans people.

**Table 1:** Summary of preclinical models used to study the effects of GAHT on transgender health. A semi-systematic approach was adopted to identify key literature in the trans health field using the key words ‘transgender’, ‘model’, ‘preclinical’, ‘GAHT’ and ‘mouse’ in PubMed and Google Scholar. These were input into the literature aggregation tool ‘Litmaps’ https://app.litmaps.com/ to identify additional articles not identified in initial search

Species, strain and type of GAHT	Study aim	Key findings	Reference
Mouse C57BL/6 J ApoE null Masculinising	To assess the effects of GAHT on atherosclerosis risk in female-to-male mice	Combined T + E2 treatment in reduced atherosclerosis plaque formation in pubertal female Ovx mice compared to either T or Veh alone, suggesting that adding low-dose E2 to GAHT after Ovx could improve cardiovascular outcomes for trans men	Goetz et al. 2018 [123]
Mouse C57BL/6JN Masculinising	To determine the effects of GAHT on reproductive function in female-to-male mice	T treatment in young adult female mice suppressed the HPG axis but did not deplete the ovarian reserve	Kinnear et al. 2019 [124]
Rat Wistar Masculinising	To determine the effects of GAHT on brain structure and metabolic profile in male-to-female rats	Estrogens are more potent drivers of brain changes in male rats than anti-androgen treatment	Gómez et al. 2020 [125]
Mouse CF-1 Masculinising	To determine if T modulates reproductive capability in female-to-male mice	In a female-to-mouse model, T treatment supressed reproductive cycling and reduced ovary size. Subsequent treatment with gonadotropic-stimulating hormone (eCG and hCG) resulted in the ovulation of fertilisable eggs thereby avoiding the need for testosterone cessation prior to ovarian stimulation	Bartels et al. 2021 [126]
Rat Wistar Masculinising	To assess the effects of GAHT on renal function and morphology in female-to-male mice	T induces renal adaptation in female rats which resembles that observed in trans men on long-term testosterone therapy	Lichtenecker et al. 2021 [127]
Mice C57BL/6 J Puberty suppression	To investigate the effects of GnRHa treatment on behaviour and brain morphology in male and female mice	GnRHa treatment after puberty onset in male and female mice exerts sex-specific effects on social- and affective behaviour, stress regulation and neural activity	Anacker et al. 2021 [128]
Mice C57BL/6N Masculinising	To determine if T-induced acyclicity can be reversed after halting T administration in female-to-male mice	T-induced amenorrhea in female mice is reversible which has implications for transgender men wishing to pause T to pursue pregnancy or oocyte donation	Kinnear et al. 2021 [129]
Pig Masculinising	To determine the effects of T on serum insulin and peripheral insulin sensitivity in female-to-male pigs	T treatment in female-to-male pigs decreases serum insulin and C-peptide concentrations whilst blood glucose and serum glycerol are not affected. These data suggest that insulin resistance in transgender men may be due to suppression of the insulin-signalling pathway and decreased insulin sensitivity in white adipose tissue	Kothmann et al. 2021 [130]
Mice TC17 C57BL/6 J Masculinising	To determine the effects of androgen excess on ovarian follicles in female-to-male mice using and inducible theca cell-specific-TC17 overexpression transgenic model TC17 converts progesterone and progenelone to androgens	Serum T was increased in TC17 mice whilst E2 was unchanged. TC17 mice had ovarian features similar to trans men including impaired folliculogenesis. These mice will be valuable tool for studying the effects of Cyp17 overexpression and hyperandrogenism in the ovary	Secchi et al. 2021 [131]
Rat Wistar Feminising	To assess the effects of E2 ethanate (E2EN) and dihydroxyprogresterone acetophenide (DHPA) on metabolic and renal outcomes in male-to-female rats	GAHT combined with E2EN/DHPA produced a feminising change in male rats without altering blood pressure and renal adaptations appeared closer to female controls	Gusmão-Silva et al. 2022 [132]
Mice C57BL/6 J Masculinising	To determine the effects and mechanisms of long-term T treatment on cardiovascular function in female-to-male mice	The vascular dysfunction observed in female-to-male mice following T treatment is mediated via a T-cell/IL-17R-dependent mechanism	Santos et al. 2022 [133]
Mice C57BL/6 J Masculinising	To determine the effect of GAHT on wound healing in female-to-male mice	T inhibits cutaneous wound healing in female-to-male mice and is characterised by increased mRNA expression of inflammatory cytokines, TNFα concentrations and macrophage proliferation in the wound	Reiche et al. 2022 [134]
Mouse C57BL/6N Puberty Suppression Masculinising	To develop a female-to-male mouse model of pubertal suppression and GAHT	One of the first models to mirror female-to-male transition in adolescence using GNRHa treatment followed by T therapy	Cruz et al. 2022 [135]
Rat Sprague–Dawley Masculinising and feminising	To establish dosing regimens and to identify biomarkers of transition for future preclinical models of transgender health	The sex-specific expression of CYP450 was suggested as a suitable biomarker to guide appropriate masculinising and feminising GAHT in preclinical models of transition	Tassinari et al. 2023 [136]

*Abbreviation: Ovx*, ovariectomy; *HPG*, hypothalamic pituitary gonadal; *GnRHa*, gonadotropin receptor hormone agonist; *T*, testosterone; *E2*, estradiol; *Veh*, vehicle; *wks*, weeks; *CA*, cyproterone acetate; *eCG*, equine chorionic gonadotropin; *hCG*, human chorionic gonadotropin; *APOE*, apolipoprotein E; *CYP17*, 17α-hydroxylase/17,20-desmolase

### The Effects of GAHT on Bone in Preclinical Mouse Models of Female-to-Male Transition

The female-to-male mouse model developed by Goetz et al. focussed on determining the long-term effects GAHT after ovariectomy on bone with transitioning commencing either at adolescence or early adulthood. Female mice were ovariectomised at 6 or 10 weeks of age and administered vehicle or testosterone (dose used in trans men adjusted for average mouse body weight, 31 µg/week) at the time of ovariectomy for 14 or 10 weeks, respectively [118]. Serum testosterone concentrations were moderately increased by 1.35- to 1.5-fold compared to ovariectomised controls, whilst serum estradiol concentrations were unaffected. These data suggest that the dose of testosterone was insufficient to exert negative feedback on the HPG axis to inhibit estradiol synthesis as observed in trans men following GAHT [120]. Alternatively, the ELISA method used lacked the sensitivity required to detect significant differences between the groups [45] evidenced by the unexpected detection of estradiol in the ovariectomised control mice. Surprisingly, testosterone treatment in young ovariectomised female-to-male mice at 6 weeks of age mice resulted in a − 35% decrease in trabecular BV/TV compared to vehicle-treated ovariectomised controls. In contrast, testosterone treatment had no effect on BV/TV in females ovariectomised at 10 weeks of age. In cortical bone, testosterone treatment increased bone area and medullary volume which resulted in a decreased in cortical bone fraction in the female-to-male mice ovariectomised at 6 but not 10 weeks of age. In the absence of intact age-matched male and female control groups, the detrimental effect of testosterone treatment on trabecular bone in the female-to-male mice ovariectomised at 6 weeks of age is difficult to interpret. Given both the pubertal female control and experimental groups were ovariectomised, one would predict that if the testosterone treatment administered was not sufficient to promote bone accrual and/or preserve the existing bone in the absence of oestrogen, then over the course of the experiment (14 weeks), both control and experimental groups would have exhibited the same degree of bone loss due to oestrogen deficiency. The exacerbated bone loss in the adolescent ovariectomised females administered testosterone is intriguing and warrants further investigation.

In a follow-up experiment, the authors examined whether supplementing the testosterone treatment with a low dose of estradiol (0.8 µg/week) could prevent the bone loss observed in the early-transition model (Ovx and GAHT at 6 weeks of age) treated with testosterone alone. Following 14 weeks of treatment, serum estradiol concentrations were increased fivefold whilst serum testosterone concentrations were unaffected. The combination of testosterone and estradiol treatment increased both BMD in the spine and femur by increasing trabecular BV/TV compared to testosterone treatment alone. These data highlight the potential benefit of supplementing GAHT in trans men who have undergone ovariectomy in adolescence with a low dose of estradiol to prevent the microstructural decay associated with oestrogen deficiency.

To address whether the timing of commencement of GAHT following pubertal suppression in trans boys impacts the effects on bone composition and accrual, Dubios et al. developed a mouse model of male-to-female transition whereby puberty was suppressed in prepubertal C57Bl/6 female mice at 4 weeks of age by GnRHa with testosterone treatment commencing at either early puberty (6 weeks of age) or late puberty (8 weeks of age) [119••]. Body composition and bone microstructure and strength were assessed at 16 weeks of age at a time when peak bone mass has been achieved and importantly, all analyses were compared to both male and female controls. GnRHa treatment in prepubertal female mice altered body composition characterised by increased body fat mass and decreased lean mass, grip strength, peak bone mass accrual and strength compared to male and controls. Independent of the time of administration, testosterone reversed the impact of GnRHa in female-to-male mice on body composition to male control levels, whilst cortical bone mass and strength were restored to female control levels. Early testosterone treatment restored the GnRHa associated deficit in trabecular bone volume to male values with late testosterone treatment reversing trabecular bone volume to female levels. Importantly, if these data are translated to humans in future studies, then commencement of GAHT in trans boys, irrespective of timing following puberty suppression, will restore bone health by reversing the negative impact of GnRHa on body composition similar to that of cis males, whilst restoring peak bone mass and strength to levels observed cis females, thereby maintaining bone health and decreasing fracture risk.

## Critical Questions Related to Transgender Bone Strength That Can Be Answered Using Preclinical Models 

In addition to the significant information, preclinical models can provide on the effects of GAHT on bone microstructure and cellular activity in both trans men and women, perhaps their greatest value is the data they can provide related to fracture risk by the ability to perform direct measures of bone elasticity and strength, which is not possible in humans. Together, understanding the changes to bone microarchitecture, bone remodelling and strength following GAHT in mice may allow inferences to be made regarding the effects of GAHT in trans people, potentially including predictions regarding fracture risk This is particularly important as given that clinical data regarding the effects of GAHT on fracture risk in trans men and trans women is limited to two studies [23, 30], whilst fracture risk in trans adolescents undergoing GnRHa and GAHT have not yet been reported.

Preclinical models also provide a unique opportunity whereby a complete understanding of the importance of aromatase activity within bone for preserving skeletal integrity in both males and females, during puberty and adulthood can be gained. This key information might then be used to personalise strategies to preserve bone health in people receiving GAHT as well as provide the basis for the development of potential new therapies targeted at increasing aromatase activity in bone. The use of preclinical models of transition may also facilitate the optimisation of GAHT in trans people by testing the effectiveness of interventions such as exercise or osteoporotic therapies in preserving skeletal integrity if required. Furthermore, preclinical models will also allow the trialling of novel compounds for GAHT such as selective oestrogen receptor modulators (SERMs) [121] or aromatase inducing agents [122]. These would not only pertinent to trans patients but also to patients with other degenerative bone disorders such as osteoporosis.

## Conclusion

It is apparent from the existing literature that despite the transgender population being a significant and increasing sector of our community, they are a poorly studied population evidenced by many clinical studies that lack pertinent controls or are performed retrospectively. The most appropriate control group for these studies would be trans individuals who are GAHT naïve but since withholding GAHT is considered unethical, recruitment of these patient cohorts is not possible. In addition, other factors including personalised treatment regimens, socioeconomic factors and barriers accessing healthcare make designing rigorous studies investigating bone health in trans peoples extremely challenging. Preclinical models offer the opportunity to provide significant insight into the fundamental aspects of the physiological actions of GAHT on bone to regulate its activity and provide the unique opportunity to answer the long-standing question in bone biology, that is, what is the contribution of circulating concentrations of sex hormones versus their local concentrations within bone in maintaining skeletal integrity? These data are essential to fully understanding the effects of GAHT on bone health in trans people. Should deleterious effects of GAHT on bone health be identified, preclinical models might prove useful to test interventions to preserve skeletal integrity and strength during GAHT. Such insights have the potential to inform human physiology and may ultimately contribute to refining clinical approaches to preserve bone and to prevent fractures in transgender people undergoing GAHT, including prescribed targeted lifestyle measures and personalised therapies which in selected cases may include osteoporotic drug therapy. Crucially, if considered together, the data generated from well-designed clinical and preclinical studies will assist with the ultimate goal of allowing individuals to transition to their desired gender without compromising their bone health.

## References

[1] van Leerdam TR, Zajac JD, Cheung AS (2023). The effect of gender-affirming hormones on gender dysphoria, quality of life, and psychological functioning in transgender individuals: a systematic review. Transgend Health.

[2] Moreira-Andres MN, Canizo FJ, de la Cruz FJ, Gomez-de la Camara A, Bone Hawkins FG. (1998). mineral status in prepubertal children with constitutional delay of growth and puberty. Eur J Endocrinol.

[3] Laurent MR, Gielen E, Orwoll E, Vanderschueren D. Osteoporosis in men: what is similar and what is different? In Dempster DW, Cauley JA, Bouxsein ML, Cosman F (eds) Marcus and Feldman’s osteoporosis. Place: Academic Press, Published, pp 589–632.

[4] Karastergiou K, Smith SR, Greenberg AS, Fried SK (2012). Sex differences in human adipose tissues - the biology of pear shape. Biol Sex Differ.

[5] Janssen I, Heymsfield SB, Wang ZM, Ross R (2000). Skeletal muscle mass and distribution in 468 men and women aged 18–88 yr. J Appl Physiol.

[6] Almeida M, Laurent MR, Dubois V, Claessens F, O’Brien CA, Bouillon R, Vanderschueren D, Manolagas SC (2017). Estrogens and androgens in skeletal physiology and pathophysiology. Physiol Rev.

[7] Klink D, Caris M, Heijboer A, van Trotsenburg M, Rotteveel J (2015). Bone mass in young adulthood following gonadotropin-releasing hormone analog treatment and cross-sex hormone treatment in adolescents with gender dysphoria. J Clin Endocrinol Metab.

[8] Stoffers IE, de Vries MC, Hannema SE (2019). Physical changes, laboratory parameters, and bone mineral density during testosterone treatment in adolescents with gender dysphoria. J Sex Med.

[9] Joseph T, Ting J, Butler G (2019). The effect of GnRH analogue treatment on bone mineral density in young adolescents with gender dysphoria: findings from a large national cohort. J Pediatr Endocrinol Metab.

[10] Lee JY, Finlayson C, Olson-Kennedy J, Garofalo R, Chan YM, Glidden DV, Rosenthal SM (2020). Low bone mineral density in early pubertal transgender/gender diverse youth: findings from the trans youth care study. J Endocr Soc.

[11] Navabi B, Tang K, Khatchadourian K, Lawson ML. Pubertal suppression, bone mass, and body composition in youth with gender dysphoria. Pediatrics. 2021;148(4). 10.1542/peds.2020-039339. 10.1542/peds.2020-039339 34497118

[12] Vlot MC, Klink DT, den Heijer M, Blankenstein MA, Rotteveel J, Heijboer AC (2017). Effect of pubertal suppression and cross-sex hormone therapy on bone turnover markers and bone mineral apparent density (BMAD) in transgender adolescents. Bone..

[13] Schagen SEE, Wouters FM, Cohen-Kettenis PT, Gooren LJ, Hannema SE (2020). Bone development in transgender adolescents treated with GnRH analogues and subsequent gender-affirming hormones. J Clin Endocrinol Metab.

[14] Delgado-Ruiz R, Swanson P, Romanos G (2019). Systematic review of the long-term effects of transgender hormone therapy on bone markers and bone mineral density and their potential effects in implant therapy. J Clin Med.

[15] Fighera TM, Ziegelmann PK, Rasia da Silva T, Spritzer PM (2019). Bone mass effects of cross-sex hormone therapy in transgender people: updated systematic review and meta-analysis. J Endocr Soc..

[16] Rothman MS, Iwamoto SJ (2019). Bone health in the transgender population. Clin Rev Bone Miner Metab.

[17] Singh-Ospina N, Maraka S, Rodriguez-Gutierrez R, Davidge-Pitts C, Nippoldt TB, Prokop LJ, Murad MH (2017). Effect of sex steroids on the bone health of transgender individuals: a systematic review and meta-analysis. J Clin Endocrinol Metab.

[18] Verroken C, Collet S, Lapauw B, T’Sjoen G (2022). Osteoporosis and bone health in transgender individuals. Calcif Tissue Int.

[19] Bretherton I, Ghasem-Zadeh A, Leemaqz SY, Seeman E, Wang X, McFarlane T, Spanos C, Grossmann M, Zajac JD, Cheung AS (2022). Bone microarchitecture in transgender adults: a cross-sectional study. J Bone Miner Res.

[20] Lips P, van Kesteren PJ, Asscheman H, Gooren LJ (1996). The effect of androgen treatment on bone metabolism in female-to-male transsexuals. J Bone Miner Res.

[21] Van Caenegem E, Wierckx K, Taes Y, Dedecker D, Van de Peer F, Toye K, Kaufman JM, T’Sjoen G (2012). Bone mass, bone geometry, and body composition in female-to-male transsexual persons after long-term cross-sex hormonal therapy. J Clin Endocrinol Metab.

[22] Lapauw B, Taes Y, Simoens S, Van Caenegem E, Weyers S, Goemaere S, Toye K, Kaufman JM, T’Sjoen GG (2008). Body composition, volumetric and areal bone parameters in male-to-female transsexual persons. Bone.

[23] Wiepjes CM, de Blok CJ, Staphorsius AS, Nota NM, Vlot MC, de Jongh RT, den Heijer M (2020). Fracture risk in trans women and trans men using long-term gender-affirming hormonal treatment: a nationwide cohort study. J Bone Miner Res.

[24] Dittrich R, Binder H, Cupisti S, Hoffmann I, Beckmann MW, Mueller A (2005). Endocrine treatment of male-to-female transsexuals using gonadotropin-releasing hormone agonist. Exp Clin Endocrinol Diabetes.

[25] Mueller A, Zollver H, Kronawitter D, Oppelt PG, Claassen T, Hoffmann I, Beckmann MW, Dittrich R (2011). Body composition and bone mineral density in male-to-female transsexuals during cross-sex hormone therapy using gonadotrophin-releasing hormone agonist. Exp Clin Endocrinol Diabetes.

[26] Wiepjes CM, Vlot MC, Klaver M, Nota NM, de Blok CJ, de Jongh RT, Lips P, Heijboer AC, Fisher AD, Schreiner T, T’Sjoen G, den Heijer M (2017). Bone mineral density increases in trans persons after 1 year of hormonal treatment: a multicenter prospective observational study. J Bone Miner Res.

[27] van Kesteren P, Lips P, Deville W, Popp-Snijders C, Asscheman H, Megens J, Gooren L (1996). The effect of one-year cross-sex hormonal treatment on bone metabolism and serum insulin-like growth factor-1 in transsexuals. J Clin Endocrinol Metab.

[28] Van Caenegem E, Wierckx K, Taes Y, Schreiner T, Vandewalle S, Toye K, Kaufman JM, T’Sjoen G (2015). Preservation of volumetric bone density and geometry in trans women during cross-sex hormonal therapy: a prospective observational study. Osteoporos Int.

[29] Wiepjes CM, de Jongh RT, de Blok CJ, Vlot MC, Lips P, Twisk JW, den Heijer M (2019). Bone safety during the first ten years of gender-affirming hormonal treatment in transwomen and transmen. J Bone Miner Res.

[30] Motta G, Marinelli L, Barale M, Brustio PR, Manieri C, Ghigo E, Procopio M, Lanfranco F (2020). Fracture risk assessment in an Italian group of transgender women after gender-confirming surgery. J Bone Miner Metab.

[31] Restar A, Dusic EJ, Garrison-Desany H, Lett E, Everhart A, Baker KE, Scheim AI, Beckham SW, Reisner S, Rose AJ, Mimiaga MJ, Radix A, Operario D, Hughto JMW. Gender affirming hormone therapy dosing behaviors among transgender and nonbinary adults. Humanit Soc Sci Commun. 2022;9(1):10.1057/s41599-022-01291-5. 10.1057/s41599-022-01291-5 PMC9833814 36636110

[32] Van Caenegem E, Taes Y, Wierckx K, Vandewalle S, Toye K, Kaufman JM, Schreiner T, Haraldsen I, T’Sjoen G (2013). Low bone mass is prevalent in male-to-female transsexual persons before the start of cross-sex hormonal therapy and gonadectomy. Bone.

[33] Giacomelli G, Meriggiola MC (2022). Bone health in transgender people: a narrative review. Ther Adv Endocrinol Metab.

[34] Cohen-Kettenis PT, Owen A, Kaijser VG, Bradley SJ, Zucker KJ (2003). Demographic characteristics, social competence, and behavior problems in children with gender identity disorder: a cross-national, cross-clinic comparative analysis. J Abnorm Child Psychol.

[35] Jardi F, Laurent MR, Kim N, Khalil R, De Bundel D, Van Eeckhaut A, Van Helleputte L, Deboel L, Dubois V, Schollaert D, Decallonne B, Carmeliet G, Van den Bosch L, D’Hooge R, Claessens F, Vanderschueren D (2018). Testosterone boosts physical activity in male mice via dopaminergic pathways. Sci Rep.

[36] Clarke MV, Russell PK, Zajac JD, Davey RA (2019). The androgen receptor in the hypothalamus positively regulates hind-limb muscle mass and voluntary physical activity in adult male mice. J Steroid Biochem Mol Biol.

[37] Cabelka CA, Baumann CW, Collins BC, Nash N, Le G, Lindsay A, Spangenburg EE, Lowe DA (2019). Effects of ovarian hormones and estrogen receptor alpha on physical activity and skeletal muscle fatigue in female mice. Exp Gerontol.

[38] Jilka RL (2013). The relevance of mouse models for investigating age-related bone loss in humans. J Gerontol A Biol Sci Med Sci.

[39] Glatt V, Canalis E, Stadmeyer L, Bouxsein ML (2007). Age-related changes in trabecular architecture differ in female and male C57BL/6J mice. J Bone Miner Res.

[40] Baron R, Tross R, Vignery A (1984). Evidence of sequential remodeling in rat trabecular bone: morphology, dynamic histomorphometry, and changes during skeletal maturation. Anat Rec.

[41] Reim NS, Breig B, Stahr K, Eberle J, Hoeflich A, Wolf E, Erben RG (2008). Cortical bone loss in androgen-deficient aged male rats is mainly caused by increased endocortical bone remodeling. J Bone Miner Res.

[42] Jilka RL, O’Brien CA (2016). The role of osteocytes in age-related bone loss. Curr Osteoporos Rep.

[43] Piemontese M, Almeida M, Robling AG, Kim HN, Xiong J, Thostenson JD, Weinstein RS, Manolagas SC, O’Brien CA, Jilka RL. Old age causes de novo intracortical bone remodeling and porosity in mice. JCI Insight. 2017;2(17):10.1172/jci.insight.93771. 10.1172/jci.insight.93771 PMC5621920 28878136

[44] Laurent MR, Hammond GL, Blokland M, Jardi F, Antonio L, Dubois V, Khalil R, Sterk SS, Gielen E, Decallonne B, Carmeliet G, Kaufman JM, Fiers T, Huhtaniemi IT, Vanderschueren D, Claessens F (2016). Sex hormone-binding globulin regulation of androgen bioactivity in vivo: validation of the free hormone hypothesis. Sci Rep..

[45] Rosner W, Hankinson SE, Sluss PM, Vesper HW, Wierman ME (2013). Challenges to the measurement of estradiol: an endocrine society position statement. J Clin Endocrinol Metab.

[46] Beamer WG, Donahue LR, Rosen CJ, Baylink DJ (1996). Genetic variability in adult bone density among inbred strains of mice. Bone.

[47] Bouxsein ML, Myers KS, Shultz KL, Donahue LR, Rosen CJ, Beamer WG (2005). Ovariectomy-induced bone loss varies among inbred strains of mice. J Bone Miner Res.

[48] Fang J, Yang L, Zhang R, Zhu X, Wang P (2015). Are there differences between Sprague-Dawley and Wistar rats in long-term effects of ovariectomy as a model for postmenopausal osteoporosis?. Int J Clin Exp Pathol.

[49] Sharma A, Michels LV, Pitsillides AA, Greeves J, Plotkin LI, Cardo V, Sims NA, Clarkin CE (2023). Sexing bones: improving transparency of sex reporting to address bias within preclinical studies. J Bone Miner Res.

[50] Nelson JF, Karelus K, Felicio LS, Johnson TE (1990). Genetic influences on the timing of puberty in mice. Biol Reprod.

[51] Zhou Y, Zhu W, Guo Z, Zhao Y, Song Z, Xiao J (2007). Effects of maternal nuclear genome on the timing of puberty in mice offspring. J Endocrinol.

[52] Bell MR (2018). Comparing postnatal development of gonadal hormones and associated social behaviors in rats, mice, and humans. Endocrinology.

[53] Lu J, Shin Y, Yen MS, Sun SS (2016). Peak bone mass and patterns of change in total bone mineral density and bone mineral contents from childhood into young adulthood. J Clin Densitom.

[54] Jackson SJ, Andrews N, Ball D, Bellantuono I, Gray J, Hachoumi L, Holmes A, Latcham J, Petrie A, Potter P, Rice A, Ritchie A, Stewart M, Strepka C, Yeoman M, Chapman K (2017). Does age matter? The impact of rodent age on study outcomes. Lab Anim.

[55] Khosla S, Monroe DG. Regulation of bone metabolism by sex steroids. Cold Spring Harb Perspect Med. 2018;8(1):10.1101/cshperspect.a031211. 10.1101/cshperspect.a031211 PMC5749141 28710257

[56] Rooney AM, van der Meulen MCH (2017). Mouse models to evaluate the role of estrogen receptor alpha in skeletal maintenance and adaptation. Ann N Y Acad Sci.

[57] Vico L, Vanacker JM (2010). Sex hormones and their receptors in bone homeostasis: insights from genetically modified mouse models. Osteoporos Int.

[58] Gurkan L, Ekeland A, Gautvik KM, Langeland N, Ronningen H, Solheim LF (1986). Bone changes after castration in rats. A model for osteoporosis. Acta Orthop Scand..

[59] Schot LP, Schuurs AH (1990). Pathophysiology of bone loss in castrated animals. J Steroid Biochem Mol Biol.

[60] van Weerden WM, Bierings HG, van Steenbrugge GJ, de Jong FH, Schroder FH (1992). Adrenal glands of mouse and rat do not synthesize androgens. Life Sci.

[61] Roberts BC, Giorgi M, Oliviero S, Wang N, Boudiffa M, Dall’Ara E (2019). The longitudinal effects of ovariectomy on the morphometric, densitometric and mechanical properties in the murine tibia: a comparison between two mouse strains. Bone..

[62] Vanderschueren D, Vandenput L, Boonen S, Lindberg MK, Bouillon R, Ohlsson C (2004). Androgens and bone. Endocr Rev.

[63] Deckard C, Walker A, Hill BJ (2017). Using three-point bending to evaluate tibia bone strength in ovariectomized young mice. J Biol Phys.

[64] Stephens M, Lopez-Linares K, Aldazabal J, Macias I, Ortuzar N, Bengoetxea H, Bulnes S, Alcorta-Sevillano N, Infante A, Lafuente JV, Rodriguez CI (2021). Murine femur micro-computed tomography and biomechanical datasets for an ovariectomy-induced osteoporosis model. Sci Data.

[65] Fonseca D, Ward WE (2004). Daidzein together with high calcium preserve bone mass and biomechanical strength at multiple sites in ovariectomized mice. Bone.

[66] Kim NR, Khalil R, David K, Antonio L, Schollaert D, Deboel L, Van Herck E, Wardenier N, Cools M, Decallonne B, Claessens F, Dubois V, Vanderschueren D (2021). Novel model to study the physiological effects of temporary or prolonged sex steroid deficiency in male mice. Am J Physiol Endocrinol Metab.

[67] Wang Y, Yano T, Kikuchi A, Yano N, Matsumi H, Ando K, Kasai Y, Watanabe M, Okagaki R, Osuga Y, Taketani Y (2000). Comparison of the effects of add-back therapy with various natural oestrogens on bone metabolism in rats administered a long-acting gonadotrophin-releasing hormone agonist. J Endocrinol.

[68] Almeida M, Iyer S, Martin-Millan M, Bartell SM, Han L, Ambrogini E, Onal M, Xiong J, Weinstein RS, Jilka RL, O’Brien CA, Manolagas SC (2013). Estrogen receptor-alpha signaling in osteoblast progenitors stimulates cortical bone accrual. J Clin Invest.

[69] Rakover Y, Lu P, Briody JN, Tao C, Weiner E, Ederveen AG, Cowell CT, Ben-Shlomo I (2000). Effects of delaying puberty on bone mineralization in female rats. Hum Reprod.

[70] Perry MJ, Gujra S, Whitworth T, Tobias JH (2005). Tamoxifen stimulates cancellous bone formation in long bones of female mice. Endocrinology.

[71] Komrakova M, Nagel J, Hoffmann DB, Lehmann W, Schilling AF, Sehmisch S (2020). Effect of selective androgen receptor modulator enobosarm on bone healing in a rat model for aged male osteoporosis. Calcif Tissue Int.

[72] Saki F, Kasaee SR, Sadeghian F, Talezadeh P, Ranjbar Omrani GH (2019). The effect of testosterone itself and in combination with letrozole on bone mineral density in male rats. J Bone Miner Metab.

[73] Ohlsson C, Engdahl C, Borjesson AE, Windahl SH, Studer E, Westberg L, Eriksson E, Koskela A, Tuukkanen J, Krust A, Chambon P, Carlsten H, Lagerquist MK (2012). Estrogen receptor-alpha expression in neuronal cells affects bone mass. Proc Natl Acad Sci U S A.

[74] Laurent MR, Jardi F, Dubois V, Schollaert D, Khalil R, Gielen E, Carmeliet G, Claessens F, Vanderschueren D (2016). Androgens have antiresorptive effects on trabecular disuse osteopenia independent from muscle atrophy. Bone.

[75] Emmanuelle NE, Marie-Cecile V, Florence T, Jean-Francois A, Francoise L, Coralie F, Alexia V (2021). Critical role of estrogens on bone homeostasis in both male and female: from physiology to medical implications. Int J Mol Sci.

[76] Arao Y, Hamilton KJ, Wu SP, Tsai MJ, DeMayo FJ, Korach KS (2019). Dysregulation of hypothalamic-pituitary estrogen receptor alpha-mediated signaling causes episodic LH secretion and cystic ovary. FASEB J.

[77] Sims NA, Clément-Lacroix P, Minet D, Fraslon-Vanhulle C, Gaillard-Kelly M, Resche-Rigon M, Baron R (2003). A functional androgen receptor is not sufficient to allow estradiol to protect bone after gonadectomy in estradiol receptor–deficient mice. J Clin Investig.

[78] Khalid AB, Krum SA (2016). Estrogen receptors alpha and beta in bone. Bone.

[79] Antal MC, Krust A, Chambon P, Mark M (2008). Sterility and absence of histopathological defects in nonreproductive organs of a mouse ERbeta-null mutant. Proc Natl Acad Sci U S A.

[80] Moverare S, Venken K, Eriksson AL, Andersson N, Skrtic S, Wergedal J, Mohan S, Salmon P, Bouillon R, Gustafsson JA, Vanderschueren D, Ohlsson C (2003). Differential effects on bone of estrogen receptor alpha and androgen receptor activation in orchidectomized adult male mice. Proc Natl Acad Sci U S A.

[81] Saxon LK, Robling AG, Castillo AB, Mohan S, Turner CH (2007). The skeletal responsiveness to mechanical loading is enhanced in mice with a null mutation in estrogen receptor-beta. Am J Physiol Endocrinol Metab.

[82] Iravani M, Lagerquist MK, Karimian E, Chagin AS, Ohlsson C, Savendahl L (2019). Effects of the selective GPER1 agonist G1 on bone growth. Endocr Connect.

[83] Chou YS, Chuang SC, Chen CH, Ho ML, Chang JK (2021). G-protein-coupled estrogen receptor-1 positively regulates the growth plate chondrocyte proliferation in female pubertal mice. Front Cell Dev Biol.

[84] Martin-Millan M, Almeida M, Ambrogini E, Han L, Zhao H, Weinstein RS, Jilka RL, O’Brien CA, Manolagas SC (2010). The estrogen receptor-alpha in osteoclasts mediates the protective effects of estrogens on cancellous but not cortical bone. Mol Endocrinol.

[85] Nakamura T, Imai Y, Matsumoto T, Sato S, Takeuchi K, Igarashi K, Harada Y, Azuma Y, Krust A, Yamamoto Y, Nishina H, Takeda S, Takayanagi H, Metzger D, Kanno J, Takaoka K, Martin TJ, Chambon P, Kato S (2007). Estrogen prevents bone loss via estrogen receptor alpha and induction of Fas ligand in osteoclasts. Cell.

[86] Maatta JA, Buki KG, Gu G, Alanne MH, Vaaraniemi J, Liljenback H, Poutanen M, Harkonen P, Vaananen K (2013). Inactivation of estrogen receptor alpha in bone-forming cells induces bone loss in female mice. FASEB J.

[87] Windahl SH, Borjesson AE, Farman HH, Engdahl C, Moverare-Skrtic S, Sjogren K, Lagerquist MK, Kindblom JM, Koskela A, Tuukkanen J, Divieti Pajevic P, Feng JQ, Dahlman-Wright K, Antonson P, Gustafsson JA, Ohlsson C (2013). Estrogen receptor-alpha in osteocytes is important for trabecular bone formation in male mice. Proc Natl Acad Sci U S A.

[88] Kondoh S, Inoue K, Igarashi K, Sugizaki H, Shirode-Fukuda Y, Inoue E, Yu T, Takeuchi JK, Kanno J, Bonewald LF, Imai Y (2014). Estrogen receptor alpha in osteocytes regulates trabecular bone formation in female mice. Bone.

[89] Doolittle ML, Saul D, Kaur J, Rowsey JL, Eckhardt B, Vos S, Grain S, Kroupova K, Ruan M, Weivoda M, Oursler MJ, Farr JN, Monroe DG, Khosla S (2022). Skeletal effects of inducible ERalpha deletion in osteocytes in adult mice. J Bone Miner Res.

[90] Zhong ZA, Sun W, Chen H, Zhang H, Lay YE, Lane NE, Yao W (2015). Optimizing tamoxifen-inducible Cre/loxp system to reduce tamoxifen effect on bone turnover in long bones of young mice. Bone..

[91] Xie Z, McGrath C, Sankaran J, Styner M, Little-Letsinger S, Dudakovic A, van Wijnen AJ, Rubin J, Sen B (2021). Low-dose tamoxifen induces significant bone formation in mice. JBMR Plus..

[92] Jardi F, Laurent MR, Dubois V, Khalil R, Deboel L, Schollaert D, Van Den Bosch L, Decallonne B, Carmeliet G, Claessens F, Vanderschueren D (2017). A shortened tamoxifen induction scheme to induce CreER recombinase without side effects on the male mouse skeleton. Mol Cell Endocrinol.

[93] Nicks KM, Fujita K, Fraser D, McGregor U, Drake MT, McGee-Lawrence ME, Westendorf JJ, Monroe DG, Khosla S (2016). Deletion of estrogen receptor beta in osteoprogenitor cells increases trabecular but not cortical bone mass in female mice. J Bone Miner Res.

[94] Xu X, Yang H, Bullock WA, Gallant MA, Ohlsson C, Bellido TM, Main RP (2023). Osteocyte estrogen receptor beta (Ot-ERbeta) regulates bone turnover and skeletal adaptive response to mechanical loading differently in male and female growing and adult mice. J Bone Miner Res.

[95] Kawano H, Sato T, Yamada T, Matsumoto T, Sekine K, Watanabe T, Nakamura T, Fukuda T, Yoshimura K, Yoshizawa T, Aihara K, Yamamoto Y, Nakamichi Y, Metzger D, Chambon P, Nakamura K, Kawaguchi H, Kato S (2003). Suppressive function of androgen receptor in bone resorption. Proc Natl Acad Sci U S A.

[96] MacLean HE, Moore AJ, Sastra SA, Morris HA, Ghasem-Zadeh A, Rana K, Axell AM, Notini AJ, Handelsman DJ, Seeman E, Zajac JD, Davey RA (2010). DNA-binding-dependent androgen receptor signaling contributes to gender differences and has physiological actions in males and females. J Endocrinol.

[97] Venken K, De Gendt K, Boonen S, Ophoff J, Bouillon R, Swinnen JV, Verhoeven G, Vanderschueren D (2006). Relative impact of androgen and estrogen receptor activation in the effects of androgens on trabecular and cortical bone in growing male mice: a study in the androgen receptor knockout mouse model. J Bone Mineral Res : Off J Am Soc Bone Mineral Res.

[98] Yeh S, Tsai MY, Xu Q, Mu XM, Lardy H, Huang KE, Lin H, Yeh SD, Altuwaijri S, Zhou X, Xing L, Boyce BF, Hung MC, Zhang S, Gan L, Chang C (2002). Generation and characterization of androgen receptor knockout (ARKO) mice: an in vivo model for the study of androgen functions in selective tissues. Proc Natl Acad Sci U S A.

[99] Wu J, Henning P, Sjogren K, Koskela A, Tuukkanen J, Moverare-Skrtic S, Ohlsson C (2019). The androgen receptor is required for maintenance of bone mass in adult male mice. Mol Cell Endocrinol.

[100] Chiang C, Chiu M, Moore AJ, Anderson PH, Ghasem-Zadeh A, McManus JF, Ma C, Seeman E, Clemens TL, Morris HA, Zajac JD, Davey RA (2009). Mineralization and bone resorption are regulated by the androgen receptor in male mice. J Bone Miner Res.

[101] Notini AJ, McManus JF, Moore A, Bouxsein M, Jimenez M, Chiu WS, Glatt V, Kream BE, Handelsman DJ, Morris HA, Zajac JD, Davey RA (2007). Osteoblast deletion of exon 3 of the androgen receptor gene results in trabecular bone loss in adult male mice. J Bone Miner Res.

[102] Wiren KM, Semirale AA, Zhang XW, Woo A, Tommasini SM, Price C, Schaffler MB, Jepsen KJ (2008). Targeting of androgen receptor in bone reveals a lack of androgen anabolic action and inhibition of osteogenesis: a model for compartment-specific androgen action in the skeleton. Bone.

[103] Wiren KM, Zhang XW, Toombs AR, Kasparcova V, Gentile MA, Harada S, Jepsen KJ (2004). Targeted overexpression of androgen receptor in osteoblasts: unexpected complex bone phenotype in growing animals. Endocrinology.

[104] Russell PK, Clarke MV, Cheong K, Anderson PH, Morris HA, Wiren KM, Zajac JD, Davey RA (2015). Androgen receptor action in osteoblasts in male mice is dependent on their stage of maturation. J Bone Miner Res.

[105] Sinnesael M, Jardi F, Deboel L, Laurent MR, Dubois V, Zajac JD, Davey RA, Carmeliet G, Claessens F, Vanderschueren D (2015). The androgen receptor has no direct antiresorptive actions in mouse osteoclasts. Mol Cell Endocrinol.

[106] Sinnesael M, Claessens F, Laurent M, Dubois V, Boonen S, Deboel L, Vanderschueren D (2012). Androgen receptor (AR) in osteocytes is important for the maintenance of male skeletal integrity: evidence from targeted AR disruption in mouse osteocytes. J Bone Mineral Res : Off J Am Soc Bone Mineral Res.

[107] Bulun SE (2014). Aromatase and estrogen receptor alpha deficiency. Fertil Steril.

[108] Oz OK, Zerwekh JE, Fisher C, Graves K, Nanu L, Millsaps R, Simpson ER (2000). Bone has a sexually dimorphic response to aromatase deficiency. J Bone Miner Res.

[109] Miyaura C, Toda K, Inada M, Ohshiba T, Matsumoto C, Okada T, Ito M, Shizuta Y, Ito A (2001). Sex- and age-related response to aromatase deficiency in bone. Biochem Biophys Res Commun.

[110] Falahati-Nini A, Riggs BL, Atkinson EJ, O’Fallon WM, Eastell R, Khosla S (2000). Relative contributions of testosterone and estrogen in regulating bone resorption and formation in normal elderly men. J Clin Invest.

[111] Finkelstein JS, Lee H, Leder BZ, Burnett-Bowie SA, Goldstein DW, Hahn CW, Hirsch SC, Linker A, Perros N, Servais AB, Taylor AP, Webb ML, Youngner JM, Yu EW (2016). Gonadal steroid-dependent effects on bone turnover and bone mineral density in men. J Clin Invest.

[112] Smith MR (2002). Osteoporosis during androgen deprivation therapy for prostate cancer. Urology..

[113] Handelsman DJ, Gibson E, Davis S, Golebiowski B, Walters KA, Desai R (2020). Ultrasensitive serum estradiol measurement by liquid chromatography-mass spectrometry in postmenopausal women and mice. J Endocr Soc..

[114] Van Caenegem E, Wierckx K, Taes Y, Schreiner T, Vandewalle S, Toye K, Lapauw B, Kaufman JM, T’Sjoen G (2015). Body composition, bone turnover, and bone mass in trans men during testosterone treatment: 1-year follow-up data from a prospective case-controlled study (ENIGI). Eur J Endocrinol / Eur Fed Endocr Soc.

[115] Sjogren K, Lagerquist M, Moverare-Skrtic S, Andersson N, Windahl SH, Swanson C, Mohan S, Poutanen M, Ohlsson C (2009). Elevated aromatase expression in osteoblasts leads to increased bone mass without systemic adverse effects. J Bone Miner Res.

[116] Kouretas D, Laliotis V, Taitzoglou I, Georgellis A, Tsantarliotou M, Mougios V, Amiridis G, Antonoglou O (1999). Sex-hormone binding globulin from sheep serum: purification and effects of pregnancy and treatment with exogenous estradiol. Comp Biochem Physiol C Pharmacol Toxicol Endocrinol.

[117] Oheim R, Schinke T, Amling M, Pogoda P (2016). Can we induce osteoporosis in animals comparable to the human situation?. Injury.

[118] Goetz TG, Mamillapalli R, Devlin MJ, Robbins AE, Majidi-Zolbin M, Taylor HS (2017). Cross-sex testosterone therapy in ovariectomized mice: addition of low-dose estrogen preserves bone architecture. Am J Physiol Endocrinol Metab.

[119] Dubois V, Ciancia S, Doms S, El Kharraz S, Sommers V, Kim NR, David K, Van Dijck J, Valle-Tenney R, Maes C, Antonio L, Decallonne B, Carmeliet G, Claessens F, Cools M, Vanderschueren D (2023). Testosterone restores body composition, bone mass, and bone strength following early puberty suppression in a mouse model mimicking the clinical strategy in trans boys. J Bone Miner Res.

[120] Turner A, Chen TC, Barber TW, Malabanan AO, Holick MF, Tangpricha V (2004). Testosterone increases bone mineral density in female-to-male transsexuals: a case series of 15 subjects. Clin Endocrinol.

[121] Gennari L, Merlotti D, Nuti R (2010). Selective estrogen receptor modulator (SERM) for the treatment of osteoporosis in postmenopausal women: focus on lasofoxifene. Clin Interv Aging..

[122] Miki Y, Hata S, Ono K, Suzuki T, Ito K, Kumamoto H, Sasano H (2017). Roles of aryl hydrocarbon receptor in aromatase-dependent cell proliferation in human osteoblasts. Int J Mol Sci.

[123] Goetz TG, Mamillapalli R, Sahin C, Majidi-Zolbin M, Ge G, Mani A, Taylor HS (2018). Addition of estradiol to cross-sex testosterone therapy reduces atherosclerosis plaque formation in female ApoE-/- mice. Endocrinology.

[124] Kinnear HM, Constance ES, David A, Marsh EE, Padmanabhan V, Shikanov A, Moravek MB (2019). A mouse model to investigate the impact of testosterone therapy on reproduction in transgender men. Hum Reprod.

[125] Gomez A, Cerdan S, Perez-Laso C, Ortega E, Pasaro E, Fernandez R, Gomez-Gil E, Mora M, Marcos A, Del Cerro MCR, Guillamon A (2020). Effects of adult male rat feminization treatments on brain morphology and metabolomic profile. Horm Behav.

[126] Bartels CB, Uliasz TF, Lestz L, Mehlmann LM (2021). Short-term testosterone use in female mice does not impair fertilizability of eggs: implications for the fertility care of transgender males. Hum Reprod.

[127] Lichtenecker DCK, Argeri R, Castro CHM, Dias-da-Silva MR, Gomes GN (2021). Cross-sex testosterone therapy modifies the renal morphology and function in female rats and might underlie increased systolic pressure. Clin Exp Pharmacol Physiol.

[128] Anacker C, Sydnor E, Chen BK, LaGamma CC, McGowan JC, Mastrodonato A, Hunsberger HC, Shores R, Dixon RS, McEwen BS, Byne W, Meyer-Bahlburg HFL, Bockting W, Ehrhardt AA, Denny CA (2021). Behavioral and neurobiological effects of GnRH agonist treatment in mice-potential implications for puberty suppression in transgender individuals. Neuropsychopharmacology.

[129] Kinnear HM, Hashim PH, Dela Cruz C, Rubenstein G, Chang FL, Nimmagadda L, Brunette MA, Padmanabhan V, Shikanov A, Moravek MB (2021). Reversibility of testosterone-induced acyclicity after testosterone cessation in a transgender mouse model. F S Sci.

[130] Kothmann KH, Jacobsen V, Laffitte E, Bromfield C, Grizzaffi M, Jarboe M, Braundmeier-Fleming AG, Bahr JM, Nowak RA, Newell-Fugate AE (2021). Virilizing doses of testosterone decrease circulating insulin levels and differentially regulate insulin signaling in liver and adipose tissue of females. Am J Physiol Endocrinol Metab.

[131] Secchi C, Belli M, Harrison TNH, Swift J, Ko C, Duleba AJ, Stupack D, Chang RJ, Shimasaki S (2021). Effect of the spatial-temporal specific theca cell Cyp17 overexpression on the reproductive phenotype of the novel TC17 mouse. J Transl Med.

[132] Gusmao-Silva JV, Lichtenecker DCK, Ferreira LGA, Gois I, Argeri R, Gomes GN, Dias-da-Silva MR (2022). Body, metabolic and renal changes following cross-sex estrogen/progestogen therapy in a rodent model simulating its use by transwomen. J Endocrinol Invest.

[133] Santos JD, Oliveira-Neto JT, Barros PR, Damasceno LEA, Lautherbach N, Assis AP, Silva CAA, Sorgi CA, Faccioli LH, Kettelhut IC, Salgado HC, Carneiro FS, Alves-Filho JC, Tostes RC (2022). Th17 cell-linked mechanisms mediate vascular dysfunction induced by testosterone in a mouse model of gender-affirming hormone therapy. Am J Physiol Heart Circ Physiol.

[134] Reiche E, Tan Y, Louis MR, Keller PR, Soares V, Schuster CR, Lu T, O’Brien Coon D (2022). A novel mouse model for investigating the effects of gender-affirming hormone therapy on surgical healing. Plast Reconstr Surg Glob Open.

[135] Dela Cruz C, Kinnear HM, Hashim PH, Wandoff A, Nimmagadda L, Chang FL, Padmanabhan V, Shikanov A, Moravek MB (2023). A mouse model mimicking gender-affirming treatment with pubertal suppression followed by testosterone in transmasculine youth. Hum Reprod.

[136] Tassinari R, Tammaro A, Lori G, Tait S, Martinelli A, Cancemi L, Frassanito P, Maranghi F (2023). Risk assessment of transgender people: development of rodent models mimicking gender-affirming hormone therapies and identification of sex-dimorphic liver genes as novel biomarkers of sex transition. Cells.

